# Comparison of heterologous xylose transporters in recombinant *Saccharomyces cerevisiae*

**DOI:** 10.1186/1754-6834-3-5

**Published:** 2010-03-17

**Authors:** David Runquist, Bärbel Hahn-Hägerdal, Peter Rådström

**Affiliations:** 1 Applied Microbiology, Lund University, PO Box 124, SE-221 00 Lund, Sweden

## Abstract

**Background:**

Baker's yeast (*Saccharomyces cerevisiae*) has been engineered for xylose utilization to enable production of fuel ethanol from lignocellulose raw material. One unresolved challenge is that *S. cerevisiae *lacks a dedicated transport system for pentose sugars, which means that xylose is transported by non-specific Hxt transporters with comparatively low transport rate and affinity for xylose.

**Results:**

In this study, we compared three heterologous xylose transporters that have recently been shown to improve xylose uptake under different experimental conditions. The transporters Gxf1, Sut1 and At5g59250 from *Candida intermedia, Pichia stipitis *and *Arabidopsis thaliana*, respectively, were expressed in isogenic strains of *S. cerevisiae *and the transport kinetics and utilization of xylose was evaluated. Expression of the Gxf1 and Sut1 transporters led to significantly increased affinity and transport rates of xylose. In batch cultivation at 4 g/L xylose concentration, improved transport kinetics led to a corresponding increase in xylose utilization, whereas no correlation could be demonstrated at xylose concentrations greater than 15 g/L. The relative contribution of native sugar transporters to the overall xylose transport capacity was also estimated during growth on glucose and xylose.

**Conclusions:**

Kinetic characterization and aerobic batch cultivation of strains expressing the Gxf1, Sut1 and At5g59250 transporters showed a direct relationship between transport kinetics and xylose growth. The Gxf1 transporter had the highest transport capacity and the highest xylose growth rate, followed by the Sut1 transporter. The range in which transport controlled the growth rate was determined to between 0 and 15 g/L xylose. The role of catabolite repression in regulation of native transporters was also confirmed by the observation that xylose transport by native *S. cerevisiae *transporters increased significantly during cultivation in xylose and at low glucose concentration.

## Background

Baker's yeast (*Saccharomyces cerevisiae*) is used for industrial production of fuel ethanol from starch (corn) and sucrose (sugar cane). Lignocellulose biomass derived from forest and agricultural byproducts has been investigated as an alternative substrate, which does not compete with food and feed production [1,2]. Lignocellulose biomass is a complex substrate composed of hexose and pentose sugar polymers. Native *S. cerevisiae *is unable to utilize pentose sugars and considerable research has been devoted to enable utilization of xylose, which is the predominant pentose sugar in lignocellulose. Xylose utilization in *S. cerevisiae *has been accomplished by expression of the enzymes xylose reductase (XR) and xylitol dehydrogenase (XDH) or by expression of xylose isomerase (XI) [3-5].

In recombinant xylose-utilizing *S. cerevisiae*, xylose is transported by non-specific hexose transporters with poor affinity for xylose [6,7]. The degree to which transport controls xylose utilization in *S. cerevisiae *is dependent on the flux in the downstream pathway and the concentration of xylose in the medium [8,9]. The first constructed xylose-utilizing *S. cerevisiae *strains were not constrained by transport because of their slow xylose catabolism [10], whereas current recombinant *S. cerevisiae *strains are transport-constrained, at least at low substrate concentrations [9]. There have been recent reports of improved transport and utilization of xylose in *S. cerevisiae *by independent expression of the heterologous xylose transporters Gxf1 [9], Sut1 [11] and At5g5920 [12] from *Candida intermedia, Pichia stipitis *and *Arabidopsis thaliana*, respectively. However, these investigations were performed using different strains and experimental setups, which limits a comparative evaluation of the transporters and their effect on xylose utilization.

In the current study, the transporters Gxf1, Sut1 and At5g5920 were expressed in isogenic strains and assessed under identical growth conditions. Xylose transport kinetics were determined and related to aerobic xylose growth at different substrate concentrations. In addition, the contribution of native *S. cerevisiae *transporters to the overall xylose transport was estimated during growth on glucose and xylose at different concentrations. Results showed a direct relationship between xylose transport kinetics and the xylose growth rate during transport-limited conditions.

## Methods

### Strains and cultivation conditions

Plasmids and *S. cerevisiae *strains used in this study are summarized in Table 1. *Escherichia coli *strain DH5α was used for subcloning and was grown on lysogeny broth (LB) agar plates supplemented with 100 mg/L ampicillin. Defined mineral medium was used for *S. cerevisiae *cultivation (carbon source (glucose or xylose); mineral salts (5 g/L (NH_4_)_2_SO_4_, 3 g/L KH_2_PO_4, _0.5 g/L MgSO_4_·7H_2_O); vitamins and trace elements [13] and buffering agent (50 mM potassium hydrogen phthalate, pH 5.5)) [14]. Yeast strains were streaked from 15% v/v glycerol stock and grown for 2 days on yeast nitrogen base (YNB) glucose plates at 30°C. A single colony was pre-grown overnight to late exponential phase in mineral medium (20 g/L glucose) and used for subsequent inoculation. Cultivation of *S. cerevisiae *was performed at 30°C.

**Table 1:** Plasmids and strains used in the current study

Strains and plasmids	Relevant genotype	Reference
**Plasmids**		
YIplac128	*LEU2*	[33]
YIpOB9	*URA3 TDH3p-XYL1(K270R)-ADH1t, PGK1p-XYL2-PGK1t*	[19]
pSUT1		Current study
pAt5g5920		Current study
YIpDR1	YIplac128 *TDH3p-GXF1-CYC1t*	[9]
YIpDR4	YIplac128 *TDH3p-SUT1-CYC1t*	Current study
YIpDR5	YIplac128 *TDH3p-At5g5920-CYC1t*	Current study
***S. cerevisiae *strains**		
TMB 3043	CEN.PK 2-1C Δgre3, *his3*::*PGK1*p-*XKS1*-*PGK1*t, *TAL1*::*PGK1*p-*TAL1*-*PGK1*t, *TKL1*::*PGK1*p-*TKL1*-*PGK1*t, *RKI*1::*PGK1*p-*RKI1*-*PGK1*t, *RPE1*::*PGK1*p-*RPE1*-*PGK1*t, *leu2, ura3*	[34]
TMB 3662	TMB 3043, *ura3*::YIpOB9	[19]
TMB 3415	TMB 3662, *leu2*::YIplac128	Current study
TMB 3416	TMB 3662, *leu2*::YIpDR1	Current study
TMB 3418	TMB 3662, *leu2*::YIpDR4	Current study
TMB 3419	TMB 3662, *leu2*::YIpDR5	Current study

### Strain construction

Standard molecular biology techniques were used for all cloning procedures [15]. Plasmid purification was performed using a commercial kit (GeneJet Plasmid Miniprep Kit; Fermentas, Vilnius, Lithuania) and DNA was extracted from agarose gels (QIAquick Gel Extraction Kit; Qiagen, Hilden, Germany). Enzymes including T4 DNA ligase, calf intestine alkaline phosphatase, DreamTaq polymerase and various restriction enzymes were obtained from Fermentas. Transformation of *E. coli *and *S. cerevisiae *was performed using the calcium chloride [16] and the lithium acetate [17] methods, respectively.

The *SUT1 *gene from *P. stipitis *and the At5g59250gene from *A. thaliana *were codon-optimized for expression in *S. cerevisiae *by using the Codon Adaptation Index (CAI) and the Java Codon Adaptation Tool (JCat) [18]. The optimized *SUT1 *and At5g59250 genes were constructed synthetically (GenScript, Piscataway, NJ, USA), resulting in plasmids pSUT1 and pAt5g59250. The *SUT1 *and At5g59250 open reading frames (ORF) were isolated from plasmids pSUT1 and pAt5g59250 using the restriction enzymes *SpeI *and *SalI *followed by gel purification. The YIpDR4 integrative plasmid was constructed by inserting the *SUT1 *ORF between the *TDH3 *promoter and the *CYC1 *terminator of the previously constructed YIpDR1 vector (Table 1). YIpDR5 was similarly constructed using the excised At5g59250 ORF. The integrative plasmids YIpDR1, YIpDR4 and YIpDR5 were linearized using *Eco*RV, and transformed into *S. cerevisiae *strain TMB 3662 [19], yielding strains TMB 3416, TMB 3418 and TMB 3419 (Table 1). The control strain TMB 3415 was constructed by integration of plasmid YIplac128, not harboring any heterologous transporter gene, in TMB 3662 (Table 1).

### Transport kinetics

Initial xylose uptake rates were determined using a previously described method [20] with minor modifications. Cells were inoculated into 1 L shake flasks in 100 mL medium (20 g/L glucose, 0.5 g/L glucose or 60 g/L xylose) at an optical density at 620 nm (OD_620_) of 0.35, and were harvested by centrifugation (5000 g for 2 minutes) after approximately two replications (OD_620 _= 1.5). The resulting cell pellet was washed in 10 mL potassium phosphate buffer 100 mM (pH 6.8), centrifuged (5000 g for 2 minutes) and resuspended in 1 mL potassium phosphate buffer. Initial uptake rates were determined at 25°C using D-[U-^14^C] labeled xylose (GE Healthcare, Buckinghamshire, UK; specific radioactivity 500-1000 cpm/nmol). The reactions were initiated by mixing 20 μL of buffered cell solution with 20 μL of radioactive substrate, giving final substrate concentrations between 3 and 150 mM. The reaction was quenched after 20 seconds by addition of 3.5 mL ice-cold 100 mM sorbitol solution. The resulting solution was immediately filtered through a 2.5 cm GF/C filter (Whatman, Maidstone, UK) and washed with 2 × 50 mL ice-cold water. Washed filters were transferred to scintillation vials containing 5 mL scintillation cocktail (Optiphase Hisafe 2; Perkin Elmer, CT, USA) and the corresponding radioactivity was measured in a liquid scintillation counter (Tri-Carb; Perkin Elmer). The baseline radioactivity was obtained in parallel measurements by adding the radioactive substrate to the cell solution after the quenching solution had been added. All measurements were performed in biological duplicate for each strain and condition. Matlab software (Matlab R2007b; The MathWorks Inc., MA, USA) was used for non-linear regression analysis of kinetic data.

### Aerobic batch cultivation

Yeast strains were inoculated in 25 mL xylose medium (60 g/L, 15 g/L or 4 g/L) in 250 mL baffled shake flasks at a starting OD_620 _of 0.2. Cells were grown at 30°C in an orbital shaker at 200 rpm. The growth rate was followed by measuring the optical density (OD) at 620 nm. All measurements were performed in biological duplicate for each investigated strain and each condition.

## Results

### Xylose transport kinetics

**Figure 1:**
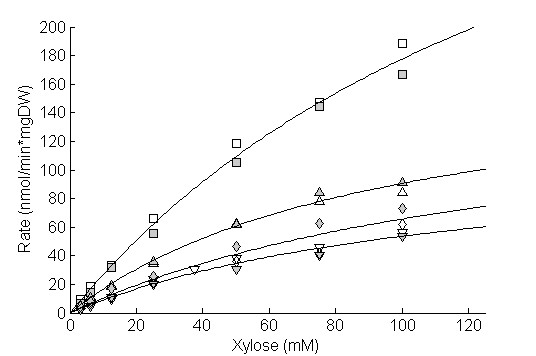
**Xylose uptake rates of *S. cerevisiae *strains expressing the Gxf1, Sut1 and At5g59250 transporters determined using D-[U^14^C] labeled sugar**. Cells were grown on 20 g/L glucose. Lines represent the calculated fit according to Michaelis-Menten kinetics. Gxf1, square; Sut1, upwards pointing triangle; At5g59250, diamond; control, downwards pointing triangle. Biological replicates are designated by empty and filled version of the same symbol.

Xylose transport kinetics were determined in the xylose-utilizing *S. cerevisiae *strain TMB 3662 [19] expressing the heterologous transporters Gxf1, Sut1 and At5g59250 from *C. intermedia, P. stipitis *and *A. thaliana*, respectively. Cells were grown in 20 g/L glucose for two cell divisions, after which xylose transport was assayed as initial rate of D-[U-^14^C] xylose uptake. The measured xylose transport rate was plotted against substrate concentration and fitted to a single-component Michaelis-Menten equation by non-linear regression (Figure 1, Table 2). Because the measured transport rate included both heterologous and native *S. cerevisiae *transporters, the determined kinetic constants refer to the overall cellular transport capacity. The ratio of the maximum transport rate, V_max _and the substrate affinity, K_m_, has, despite some shortfalls been widely used as an overall measure of enzymatic performance [21,22], and was used here for quantitative comparison of the different transporters (Table 2). Expression of the *C. intermedia *Gxf1 transporter and the *P. stipitis *Sut1 transporter was thus seen to increase the transport performance compared with the reference strain expressing only native transporters (Figure 1, Table 2). In particular, the Gxf1 transporter increased the overall xylose transport performance by three times with respect to the control strain, which is comparable with results from previous studies [9,23]. A slight improvement was observed for the strain expressing the At5g59250 transporter, but it was not statistically significant (Figure 1, Table 2).

**Table 2:** Apparent kinetic constants for xylose transport in *S. cerevisiae *strains grown on 20 g/L glucose

	**K**_**m**_**, mM**	**V**_**max**_**, nmol/min × mgDW**	**V**_**max**_**/K**_**m, **_**min**^**-1**^** × mgDW**^**-1**^
TMB 3415 (control)	121 ± 44	119 ± 17	10 × 10-^7^
TMB 3416 (Gxf1)	166 ± 6	471 ± 10	28 × 10-^7^
TMB 3418 (Sut1)	96 ± 11	178 ± 16	19 × 10-^7^
TMB 3419 (At5g59250)	148 ± 39	163 ± 7	11 × 10-^7^

### Aerobic growth rates

It has previously been shown that the effect of increased transport performance on xylose utilization is a function of strain background and substrate concentration [8,9,24]. In the current study, isogenic strains expressing the Gxf1, Sut1 and At5g59250 transporters were used to relate the measured transport kinetics to growth performance. The aerobic xylose growth rate of strains expressing the heterologous transporters was thus investigated at different substrate concentrations.

**Figure 2:**
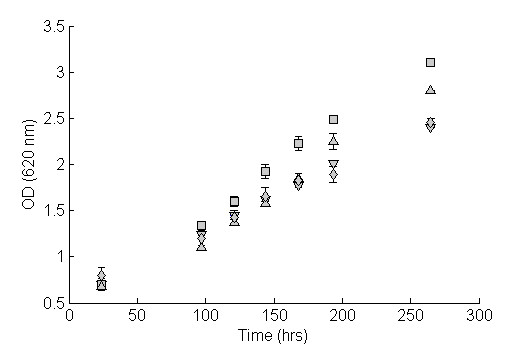
**Aerobic batch cultivation xylose 4 g/L of recombinant *S. cerevisiae *strains expressing the Gxf1, Sut1 and At5g59250 transporters**. Gxf1, square; Sut1, upwards pointing triangle; At5g59250, diamond; Control, downwards pointing triangle. Deviations between biological replicates are indicated by error bars.

Strains expressing the Gxf1, Sut1 and At5g59250 transporters were first grown at high xylose concentration (60 g/L). At this concentration, the strains expressing heterologous transporters and the control strain all displayed the same specific growth rate (μ), 0.16 h-^1^. Similarly, at intermediate xylose concentration (15 g/L), all strains still displayed the same growth rate, but it was reduced to 0.04 h-^1^. At low xylose concentration (4 g/L), the specific growth rate was further reduced to 0.002 to 0.008 h-^1^. However, at this concentration, strains expressing the heterologous transporters grew significantly faster than the control strain (Figure 2). The strain expressing the Gxf1 transporter displayed the highest growth rate, while the strain expressing the Sut1 transporter showed a small but reproducible increase in growth rate. The growth rate of the strain expressing the At5g59250 transporter was the same as that of the control strain (Figure 2).

### Xylose transport by native *S. cerevisiae* transporters

Kinetic characterization of *S. cerevisiae *strains expressing the Gxf1 and Sut1 transporters showed increased xylose transport performance compared with the control strain expressing only native transporters (Figure 1, Table 2). The performance of native *S. cerevisiae *transporters, as opposed to constitutively expressed heterologous transporters, depends on the type of transporters expressed in the cell, which in turn depends on the carbon source and its concentration [7,25]. The reported overall xylose transport affinity of native *S. cerevisiae *thus varies between 80 and 200 mM, depending on the cultivation conditions in the particular experiment [6,7,10]. In the current study, the effect of the cultivation condition was assayed by measuring the xylose transport kinetics of native *S. cerevisiae *transporters during cultivation on different concentrations of glucose and xylose.

**Figure 3:**
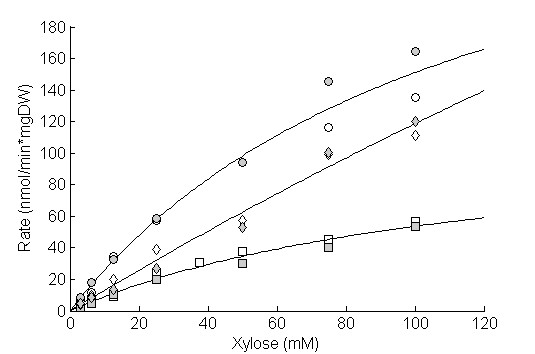
**Xylose uptake rates of *S. cerevisiae *grown under different conditions**. Lines represent the calculated fit according to Michaelis-Menten kinetics. 20 g/L Glucose, square; 0.5 g/L glucose, circle; 60 g/L xylose, diamond. Biological replicates are designated by empty and filled version of the same symbol.

TMB 3415, expressing only native transporters, was grown on 0.5 g/L and 20 g/L glucose and on 60 g/L xylose, and was subsequently assayed for xylose transport (Figure 3). The xylose transport performance (V_max_/K_m_) increased when cells were grown at 0.5 g/L glucose (28 × 10-^7 ^min-^1 ^× mgDW-^1^) and 60 g/L xylose (13 × 10-^7 ^min-^1 ^× mgDW-^1^) compared with 20 g/L glucose (10 × 10-^7 ^min-^1 ^× mgDW-^1^). Cultivation at the low glucose concentration (0.5 g/L) yielded the highest xylose transport performance, which was comparable with cells expressing the Gxf1 transporter during cultivation on 20 g/L glucose (Figure 1, Table 2). The results confirm those of previous studies, which showed increased expression of high affinity transporters and increased xylose transport during non-repressive conditions; that is, low glucose concentration [7,25].

## Discussion

Compared with glucose, the transport affinity for xylose in *S. cerevisiae *is very low [6,7]. It has thus been hypothesized that transport may control xylose utilization in recombinant *S. cerevisiae *[10]; however, the degree to which transport controls the xylose uptake rate is dependent on both the substrate concentration in the medium and the downstream pathway. Prolonged chemostat cultivation at low xylose concentration selected for increased transport affinity [26], whereas transport had a negligible effect on xylose utilization at > 10 g/L of xylose for most strains [8-10]. For this reason, many studies have failed to observe a physiological effect from overexpressing native or heterologous transporters [6,27]. Recently however, expression of xylose transporters from *C. intermedia, P. stipitis *and *A. thaliana *was reported to improve xylose utilization in recombinant *S. cerevisiae *[9,11,12]; however, different experimental designs and genetic constructions in these investigations limited a functional comparison of the results. For instance, expression of the Sut1 [11] and At5g59250 transporters [12] showed enhanced utilization rate of glucose and xylose in batch culture, whereas expression of the Gxf1 transporter [9] improved xylose utilization only. For the Gxf1 transporter, improvement of xylose utilization was also restricted to substrate concentrations > 10 g/L [9], whereas the Sut1 and At5g59250 transporters increased xylose uptake at substrate concentrations > 50 g/L [11,12].

In the current study, expression of the Gxf1, Sut1 and At5g59250 transporters was compared in isogenic strains under identical conditions. Although expression of both the Gxf1 and the Sut1 transporter increased the xylose transport performance, the effect of the Gxf1 transporter was significantly more pronounced (Figure 1. Table 2). In aerobic xylose cultivation at high and intermediate xylose concentrations (60 g/L and 15 g/L xylose, respectively) none of the heterologous transporters increased the growth rate. The results confirm the role of extracellular xylose concentration in controlling the rate of xylose transport [8,9]. Furthermore, none of the transporters influenced the growth on glucose (results not shown), which was expected because glucose transport only controls the glycolytic rate at low glucose concentration [28]. However, during aerobic cultivation at 4 g/L xylose, the measured transport kinetics were mirrored, in the sense that expression of the Gxf1 transporter led to the largest increase in growth rate, followed by the Sut1 transporter (Figure 2). Expression of the At5g59250 transporter did not increase xylose affinity and consequently the xylose growth rate was not affected (Figure 1, Figure 2).

The overall xylose transport performance of native *S. cerevisiae *was dependent on carbon source and concentration, and was highest under non-repressive conditions (xylose or low glucose) (Figure 3). Expression of native transporters clearly increased at low glucose concentrations relative to the constitutive expression of the heterologous transporters (Figure 1, Figure 3). The overall xylose transport in *S. cerevisiae *has been modeled *in silico *as a function of the extracellular glucose concentration [29]. In agreement with the current study, it was found that xylose transport was highest when the glucose concentration was ~ 0.2-1 g/L. The influence of glucose on the regulation of xylose transport has similarly been demonstrated in simultaneous saccharification and fermentation, in which a low but steady glucose concentration is maintained during xylose utilization [30]. The industrial potential of expressing heterologous xylose transporters in *S. cerevisiae *is thus highly dependent on the concentration of both xylose and glucose in the process. For example, during batch operation the concentration of glucose and xylose are high and overexpression of repressed high-affinity native transporters may increase utilization of xylose. On the other hand, during fed-batch or continuous cultivation, the concentrations of glucose and xylose are low, which indicates that native high-affinity transporters are induced and expression of a heterologous transporter with superior xylose kinetics is needed to improve uptake.

Among the transporters investigated in this work, the Gxf1 transporter clearly improved the xylose transport capacity and growth rate more than the Sut1 and At5g59250 transporters (Figure 1, Table 2, Figure 2). An explanation for these results may be found in the way these transporters were originally isolated. The At5g59250 transporter was identified solely by sequence homology to known transporter genes and thus lacked a functional screening step [12]. On the other hand, the Sut1 transporter was identified by expression of a *P. stipitis *cDNA library in a *S. cerevisiae HXT *null strain and subsequent reconstitution of growth on glucose [31]. By contrast, the Gxf1 transporter was isolated by comparing xylose growth in native xylose-utilizing yeast, including the aforementioned *P. stipitis *[32]. The highest xylose uptake was seen in the yeast *C. intermedia*, and subsequent screening of a *C. intermedia *cDNA library in an *S. cerevisiae HXT *null strain identified the *GXF1 *transporter gene [23]. Thus, the superior performance of the Gxf1 transporter demonstrated in the current study can probably be attributed to the original isolation process, which not only selected for functional expression in *S. cerevisiae *but also the highest transport capacity.

## Conclusions

In the current study, the transporters Gxf1, Sut1 and At5g59250 were compared in isogenic *S. cerevisiae *strains under identical conditions. A direct relationship was seen between transport kinetics of the individual transporters and xylose utilization under transport limiting growth conditions. The relative xylose transport performance of native *S. cerevisiae *transporters was also found to be significantly increased during cultivation at low glucose concentrations, which points towards the importance of designing an appropriate cofermentation strategy for industrial ethanol production from lignocellulose biomass.

## Competing interests

The authors declare that they have no competing interests.

## Authors' contributions

DR participated in the design of the study, performed the experimental work and wrote the manuscript. BHH participated in the design of the study and commented on the manuscript. PR participated in the design of the study and commented on the manuscript. All the authors have read and approved the final manuscript.
